# Liposomal factor VIII as an efficient pharmaceutical system for the treatment of hemophilia

**DOI:** 10.22038/IJBMS.2024.74673.16214

**Published:** 2024

**Authors:** Maryam Karimi, Seyed Mahdi Rezayat, Seyed Alireza Mortazavi, Azadeh Haeri, Mahmoud Reza Jaafari

**Affiliations:** 1 Department of Pharmaceutics and Pharmaceutical Nanotechnology, School of Pharmacy, Shahid Beheshti University of Medical Sciences, Tehran, Iran; 2 Nanotechnology Research Center, Pharmaceutical Technology Institute, Mashhad University of Medical Sciences, Mashhad, Iran; 3 Department of Medical Nanotechnology, School of Advanced Technologies in Medicine, Tehran University of Medical Sciences, Tehran, Iran; 4 Protein Technology Research Center, Shahid Beheshti University of Medical Sciences, Tehran, Iran; 5 Department of Pharmaceutical Nanotechnology, School of Pharmacy, Mashhad University of Medical Sciences, Mashhad, Iran

**Keywords:** Antihemophilic factor, Hemophilia A, Haemophilia therapy, PEGylated liposomes, Recombinant FVIII

## Abstract

**Objective(s):**

Currently, the most important treatment approach for hemophilia type A is recombinant Factor VIII. However, due to its low retention time in the blood, the patients usually need successive injections. In addition, neutralization of injected proteins by antibodies complicates treatment. We examined the prolongation of the persistence time of injectable FVIII in the blood and the potential effects on survival using promising PEGylated liposomes (PEGLip) utilizing hydrogenated soy phosphatidylcholine (HSPC, Tm= 54.5 ºC) and 1-palmitoyl-2-oleoyl-phosphatidylcholine (POPC, Tm= – 2 ºC).

**Materials and Methods:**

Nanoliposomes with different percentages of PEG (3% and 5%) were obtained via the thin film hydration procedure and extrusion. Liposomal FVIII formulation was prepared and characterization was done.

**Results:**

The results revealed that the formulations are in the 80–120 nm range with uniform dispersion, which was confirmed using transmission electron microscopy (TEM) imaging. The phase transition temperature (Tm) of the liposomes was obtained by differential scanning calorimetry (DSC). With an attachment efficacy of approximately 87%, proteins bind non-covalently yet with a strong affinity to the exterior of PEGLip. The final formulations underwent additional examination. No significant change was observed in size, charge, and PDI between the FVIII-conjugated liposomal formulations and their liposomal nanoparticles. The selected formulations were injected into BALB/c mice. The circulation time and potential clotting effectiveness of PEGLip-FVIII are vastly improved over free protein, in non-hemophilic mice.

**Conclusion:**

The obtained results showed that using phospholipids with high Tm (HSPC) can improve the hemostatic efficiency of liposomes more than phospholipids with low Tm (POPC).

## Introduction

Hemophilia A related to coagulation factor VIII, primarily affects men in a hereditary manner (1, 2). The disease manifests in severe forms in 40% of these individuals, who have spontaneously severe hemorrhagic signs in the joints, soft tissues, and various organs. This painful bleeding is potentially life-threatening, impacting the patient’s quality of life (3). Importantly, an arrangement of routine infusions keep the coagulation FVIII concentration level sufficiently elevated to avoid the mortality and morbidity linked to recurrent bleeding resulting from hemophilia (4, 5). 

The preferred drug for people with hemophilia A is intravenous recombinant coagulation factor (6). Factor VIII, with 2332 amino acids, represents a crucial plasma prominent glycoprotein in the coagulation cascade (7, 8). Because the approximate half-life of recombinant FVIII is 10 to 12 hr, efficient preventive treatment typically, requires FVIII infusions to be repeated every two to three days. Consecutive injections significantly reduce quality of life (9-12). Also, since FVIII is highly immunogenic and roughly 40% of type A patients create an inhibitory antibody response against the protein, treatment can be complicated by producing antibodies that prevent the injected protein’s action (13, 14). Consequently, a long-acting, safe form of FVIII is desperately required for therapeutic effectiveness.

Longer-acting FVIII formulations can provide prolonged bleeding protection by preventing spontaneous bleeding for more extended periods among injections compared to standard FVIII products. Therefore, they reduce the patient’s need for abrasive injections. (15-17). 

Among different nanocarriers, liposomes have several notable benefits, such as prolonged circulation time, simple fictionalization, high biocompatibility, safety, and enhanced pharmacokinetic properties of free drugs (18-21). Modifying the liposome surface with polyethylene glycol molecules avoids vesicle aggregation, increases the stability of formulations, and prevents their phagocytosis by reticuloendothelial cells (22-24). Unlike the approaches presented for producing a long-acting form of FVIII so far, the PEGLip technology does not need covalent attachment. Therefore, no changes are made in the amino acid sequence (25).

**Figure 1:**
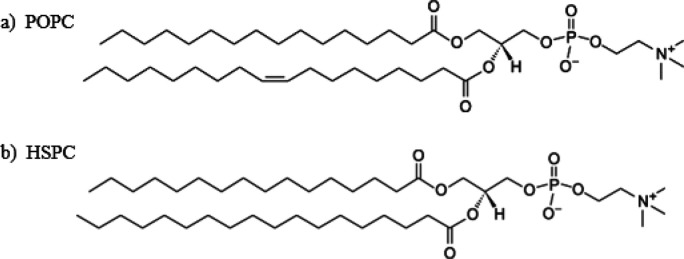
Structure of (a) 1-palmitoyl-2-oleoyl-phosphatidylcholine and (b) hydrogenated soy phosphatidylcholine

Formulating an FVIII protein with PEGylated liposomes is gentle and can be easily scaled up. The lyophilized powder of FVIII can be reconstituted in liposomal dispersion and be allowed to dissolve completely. The formulation is mild, and no covalent bonds are formed with the protein. Therefore, the native structure and biological properties of the FVIII protein do not change. They become active on the outer surface of liposomes immediately after formulation. They are free to engage with binding partners with minimum antibody generation (26-28). FVIII attaches a high specificity and affinity to the surface of PEGLip; however, this connection does not affect the three-dimensional structure or activity of the FVIII protein. *In vivo*, the hemostatic effect of PEGLip formulation comprising protein FVIII (r-FVIII or pd-FVIII) is increased. The improved survival of hemophilic mice was revealed after a tail-vein transaction. Compared to mice given normal FVIII, PEGLip-FVIII-treated mice bled less and lived noticeably longer (25, 29-31). Multiple clinical trials used a formulation made of POPC (Figure 1a) and DSPE-mPEG2000 at a 97:3 molar ratio to investigate the efficacy and safety of PEGylated FVIII. In those with critical hemophilia A, liposomal rFVIII appeared to double the number of days without a bleed compared to standard rFVIII (25, 29). 

The major objective of the research was to create an effective and stable liposomal FVIII formulation to enhance its therapeutic benefits. In order to accomplish this objective, stealth liposomes were prepared using varying percentages of mPEG (3% and 5%). Additionally, a more stable liposomal formulation was created by incorporating HSPC lipids with a high phase transition temperature (54.5 °C) (Figure 1b) (20). The complex was characterized *in vitro*, and formulation constancy was examined. The PEGylated-FVIII complex’s *in vivo* studies have been investigated in mice. We showed that in non-hemophilic mice, the circulation times are significantly prolonged. With HSPC, the hemostatic efficiency of PEGylated FVIII is dramatically increased compared to free FVIII’s.

## Materials and Methods

***Materials***

DSPE-mPEG2000, POPC, and HSPC were acquired from Lipoid (Ludwigshafen, Germany) and stored at ^_ ^20 °C. Dextran (average mol. wt. 40000), methanol, chloroform, potassium dihydrogen phosphate (KH_2_PO_4_), citric acid monohydrate (C_6_H_8_O_7·_H_2_O), ammonium molybdic acid (NH_4_)_6_Mo_7_O_24_, hydrogen peroxide (H_2_O_2_), sodium hydroxide (NaOH), hydrogen chloride (HCl), trisodium citrate (Na_3_C_6_H_5_O_7_), sulfuric acid (H_2_SO_4_), ascorbic acid (C_6_H_8_O_6_), and boric acid (H_3_BO_3_) were procured from Sigma-Aldrich company (St. Louis, MO, USA). 

***Methods***

*Formulation of nanoliposomes and* *sterilization*

Briefly (32), POPC or HSPC and DSPE-mPEG 2000 (97:3 or 95:5 mole ratio, correspondingly) were dissolved in a certain amount of chloroform in a round-bottomed flask. By connecting a rotary evaporator, after 3 hr, a thin deposit was formed. The trace of chloroform was removed using freeze-drying, after 5 hr. The formed film was hydrated using a buffer at 65 °C and argon gas until it entirely dissolved. The size of the multilamellar vesicles was subsequently decreased by sequentially extruding them via 1000–100 nm polycarbonate membrane 11 times until the final diameter of 80–120 nm was obtained. Liposomes were sterilized using a 0.22 μm Whatman membrane sterilizing filter before storage in a refrigerator. 

***Characterization of nanoliposomes***

*Zeta potential, particle size, and polydispersity index(PDI)*

Zetasizer ZS (Malvern, UK) employed Dynamic Light Scattering to assess zeta potential, PDI, and particle size. A 4-morpholine propane sulfuric acid 10 mM buffer with pH _= _7.4 was chosen to measure zeta potential. Also, dilution of liposomes during size measurement was done using 5% dextrose. The measurements were made three times at room temperature for each specimen, and their means were calculated.

*Phospholipid content assay*

According to Bartlett’s method (33), the standards were made using K_2_HPO_4_ (Dipotassium phosphate, 0.6 mmol) with phosphorus concentrations extending from 30 to 150 nmol. Then, 400 μl of sulfuric acid (10 N) was added to all special glass tubes. About 80 nmol of liposomes were poured into the sample tubes. The tubes were heated for 90 min on an aluminum block heater at 200 ^o^C. After allowing each tube to cool to ambient temperature, 100 μl H_2_O_2_) 10% v/v in deionized water( was added and heated for 5 min. Following cooling 4700 ml hexaammonium molybdate tetrahydrate (1.78 mM) produced in 0.25 N sulfuric acid, 500 μl ascorbate (0.1 g/ml) were added. Afterward, the tubes were put in a bain-marie (100^ o^C). After 20 min, with the appearance of blue color inside the tubes, the spectrophotometer was used to check the absorbance at 800 nm. 

*Negative staining of nanoliposomes for transmission electron microscopy*

In order to evaluate the morphology of nanoparticles, TEM was used (LEO 910). This approach included a 1:50 dilution of the liposomes with 5% dextrose (34). Ten microliters of liposomes were pipetted onto copper grids (Cu grids) with carbon films for 30 sec. After removing extra liposomes with filter paper, uranium acetate 2% stain (10 μl) was pipetted onto Cu grids treated with carbon within 30 sec. The samples were examined at a voltage of 80 kV after removing the excess stain with filter paper. 

*Evaluation of differential scanning calorimetry*

The thermosensitivity of liposomes is measured using DSC analysis data. In this approach, using a DSC823e calorimeter (Mettler Toledo, Switzerland), with standard aluminum pans, and an empty pan as the reference, the phase transition temperature of formulations was measured. For HSPC/DSPE-mPEG2000 (97:3) (a) and POPC/DSPE-mPEG2000 (97:3) (b), the temperature with heating speed 1 °C/min increased from -20 °C to 70 °C and _+_ 20 °C to 70 °C, correspondingly. Mettler Toledo STARe (V9.01) software was utilized to transform the raw information into molar heat capacity (MHC) (35). An examination of the 9.5 mg sample was carried out. 

*Stability of formulations*

Visual inspections and physical stability testing of the particles were done to determine the stability of PEGylated liposomes at 4 °C. To accomplish this, samples of liposomes were routinely collected (0, 4, 12, 24, and 36 weeks after preparation) and evaluated in terms of indications of creaming or sedimentation, color changes in formulas, particle size, and zeta potentials, as well as alterations in the mean particle size with time, as determined using the Malvern Nano ZS system.

*Formulation of liposomal rFVIII*

PEGLip was formulated with rFVIII as follows: 1 ml of nanoliposomes was added to a vial of 100 units of protein. Using an SRT1 roller mixer, the vial was incubated for 20 min at room temperature.

*Purification of liposomal formulation of free protein*

As earlier reported, to determine the quantity of protein linked to PEGylated liposomes, the free proteins were separated using a dextran density gradient (36). At first, 500 µl of the liposomal FVIII combination was poured into a 5-ml polypropene tube. Then 1 and 3 ml dextran (in Tris buffer) were added, 20% and 10% (w/v), respectively. The tube was placed in a Beckman ultracentrifugation SW 50.1 rotor for 30 min at 45,000 rpm (~189040 g). The unassociated protein stayed at the bottom while the liposomes and related proteins transferred to the highest part of the tube.

*Protein activity assays*

The COATEST® SP4 FVIII kit was used to carry out protein chromogenic tests following the manufacturer’s instructions.

*Drug attachment efficiency*

The distinction between the activity quantity of total protein and the associated protein in a centrifuge tube was used to determine the attachment efficiency of the protein. After separating the free proteins from the formulation using ultracentrifuge, as mentioned before, the activity level of free proteins was measured in the lower part of the tube. On the other hand, the activity level of FVIII attached to the surface of nanoliposomes was also measured in the upper part of the centrifuge tube. The efficiency of protein attachment to liposomes was calculated as follows:

Attachment efficiency (AE) % = (Associated protein activity / Total factor VIII activity) ×100

*In vivo studies*

The National Institutes of Health’s guidebook for handling and applying animals (NIH Publications No. 8023, amended 1978) was followed throughout all animal operations. According to Institutional Animal Care and Use Committee, Research Advisory Committee, and Institutional Ethical Committee norms, (IR.MUMS.AEC.1402.043) at Mashhad University of Medical Sciences, animal research was approved. Adult male BALB/c mice (19-22 g) were employed for the investigations (8–12 weeks old). They were given only an intravenous injection of liposomal rFVIII and standard rFVIII via their tail veins. Blood samples were obtained 20 min, 4, 24, and 48 hr post-administration via retro-orbital sinus (n _=_ 5 mice/ time point) and were transferred to acid-citrate dextrose tubes. After five min of centrifugation at 5000 g, plasma separation was done at 4 °C and kept at -70 °C temperature for further tests. The COATEST SP4 FVIII kit was used to perform factor VIII chromogenic tests on plasma samples per the producer’s instructions.

***Statistical analysis***

The statistical data using GraphPad Prism were assessed (version 6.00, San Diego, CA, USA). The standard deviation or standard error of the mean ± (SEM) was applied to the data. The statistics were assessed as significant at* P*<0.05 values.

## Results

*Physico-chemical characterization of nanoparticle*

In this study, liposomal formulations of POPC/DSPE-mPEG2000 and HSPC/DSPE-mPEG2000 with different percentages of DSPE-mPEG2000 (3% and 5%) were prepared. To make sure the liposomal FVIII formulation had an appropriate diameter for coagulation impacts in blood, we investigated the physicochemical characteristics of nanoliposomes in terms of zeta potential, polydispersity index (PDI), and size before and after attachment of the protein (Table 1). 

*Interaction of rFVIII with nanoliposomes*

**Figure 2:**
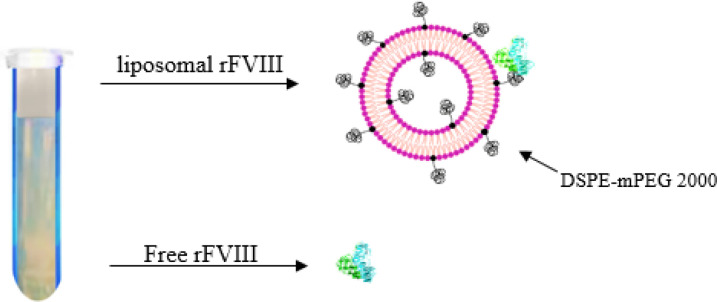
Separation of free protein from the liposomal formulation

After removing the free proteins from the liposomal formulation with the help of the dextran density gradient, the attachment efficiency (AE%) of the protein to the external surface of the nanoliposome was calculated (Figure 2). Liposomes made of HSPC/DSPE- mPEG2000 were linked to 87.1% of the added rFVIII, while those made of POPC/DSPE- mPEG2000 were linked to 86.6% approximately. The variations in mean attachment efficiencies amongst liposomal formulations are presented in Table 1 and were statistically insignificant (*P*>0.05). 

*Transmission electron microscopy (TEM) by negative staining*

**Figure 3:**
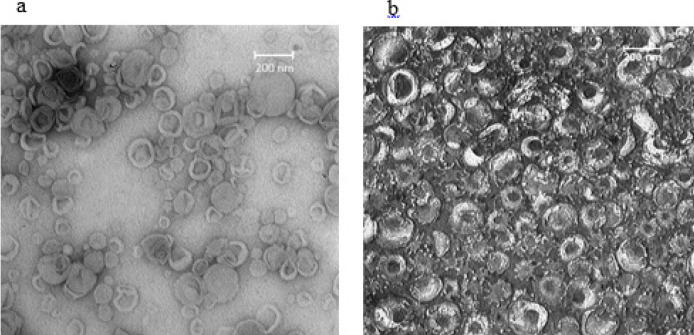
TEM images of negatively stained formulations. (a) Plain nanoliposomes (F1-a) and (b)Liposomal FVIII (F1-b)

Employing TEM, the morphological characteristics of (HSPC/DSPE-mPEG2000) and (HSPC/DSPE-mPEG2000)-rFVIII formulations were identified. Both formulations were shown to have nearly spherical forms, homogenous sizes of about 100 nm, and were essentially uniform (Figure 3). These characteristics were consistent with the outcomes of the DLS (Table 1).

*Differential scanning calorimetry*

**Figure 4:**
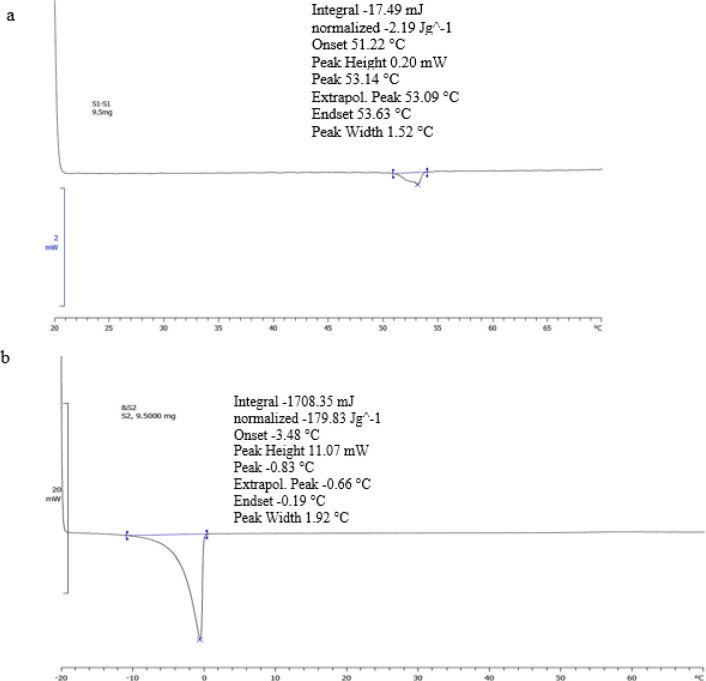
Differential scanning calorimetric analysis of liposomes 9.5 mg/ml, composed of (a) HSPC/DSPE-mPEG2000 (97:3) and (b) POPC/DSPE-mPEG2000 (97:3)

The liposomal formulations were analyzed by DSC. As shown in the thermograms of Figure 4a, it can be observed that the liposomal formulation of HSPC/DSPE-mPEG2000 showed a sharp phase transition at 53.14 °C with broadness of the peak from 51 °C to 55 °C. For liposomes composed of POPC/DSPE-mPEG2000, the sharp phase transition was found to be -0.66 °C with broadness of the peak from -3.48 °C to -0.19 °C (Figure 4b).

*Stability tests*

In the present investigation, the physical stability of POPC/DSPE-mPEG2000 and HSPC/DSPE-mPEG2000 liposomal formulations with varying percentages of DSPE-mPEG2000 was evaluated during 36 weeks at 4 °C. The formulations were analyzed in terms of appearance, size, zeta potential, and PDI. Table 2 shows that up to 36 weeks, there were no discernible variations between the four formulations’ Z-average size and Zeta potential, and only a gradual rise was noticed in these parameters. Furthermore, after 36 weeks of storage at 4 °C, the appearance of the formulations did not alter much, indicating that they were stable.

*In vivo studies*

*Biological activity assay of FVIII*

**Figure 5:**
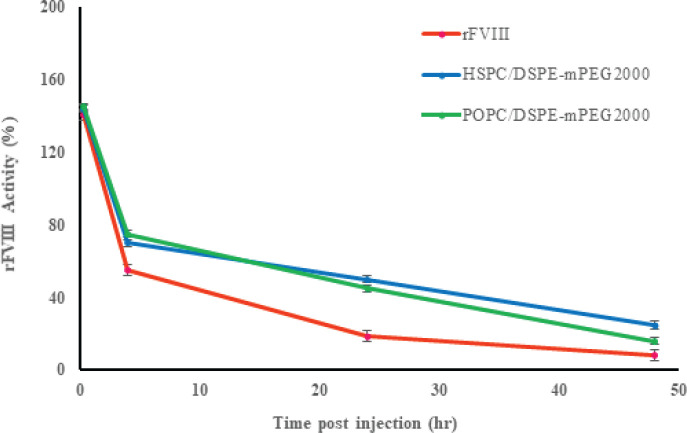
rFVIII activity levels in the presence (Blue line) of HSPC/DSPE-mPEG2000 (97:3) and (Green line) POPC/DSPE-mPEG2000 (97:3) and the absence (Orange line) of nanoliposomes following s.c. administration. Error bars indicate standard deviation

According to the earlier investigation, recombinant FVIII in POPC/DSPE-mPEG2000 (97:3) liposomal formulation prolongs circulation time in mice with hemophilia. We examined the similarity of the effects of the formulations of (HSPC/DSPE-mPEG2000)-rFVIII and (POPC/DSPE-mPEG2000)-rFVIII. Therefore, we used both formulations with percentages equal to DSPE-mPEG2000 (3%). Animal studies were conducted for PEGLip-FVIII including (HSPC/DSPE-mPEG2000)-rFVIII, (POPC/DSPE-mPEG2000)-rFVIII, and free FVIII, following administration to BALB/c mice. We administered free rFVIII and PEGLip-rFVIII injections to non-hemophilic mice’s tail veins. Three mice were used each time. The blood samples were used to determine the amount of FVIII activity in mouse plasma using a chromogenic test (Figure5).

## Discussion

As listed in Table 1, the HSPC/DSPE-mPEG2000 liposome (F1-a) size was 98.09 ± 2.1 nm with PDI of 0.14 ± 0.01, which indicates a homogeneous population of liposomes and zeta potential of -6.71 ± 0.1 mV before attachment to protein, which partly increased to 98.48 ± 0.1 nm with PDI of 0.14 ± 0.01 and zeta potential of -6.11 ± 0.1 mV after the attachment of factor VIII (*P*>0.05). The zeta average and sizes did not alter statistically significantly across formulations; however, this small size rise would indicate that protein has been conjugated to the liposomes’ surface. Similar trends were additionally confirmed in other formulations. Because PEGylated nanoliposomes can easily escape from the Reticuloendothelial System (RES) due to their relatively small surface charges, the prepared formulations are very suitable for intravenous injection.

The outcomes of rFVIII’s association with nanoliposomes were consistent with other studies that showed a very high relative affinity of FVIII for binding PEGylated nanoliposomes, and they indicate that rFVIII’s engagement with developed bilayers was persistent and not easily reversible.

*In vivo* studies indicate that the activity quantity of liposomal FVIII was higher than free protein at more than 24 hr post-injection. Also, the findings show that the use of the HSPC/DSPE-mPEG2000 liposome formulation with a high transition temperature compared to the POPC/DSPE-mPEG2000 formulation with a lower Tm shows better results after 24 hr. The highest level of FVIII activity was related to this formulation (HSPC/DSPE-mPEG2000). Immediately post-injection, FVIII activity was observed in both free and liposomal FVIII groups, indicating that the regained FVIII activity was unaffected by the external binding of FVIII to PEGLip (Figure 5). The findings of the *in vitro* assay demonstrate a strong correlation with this (Table 1).

As expected, PEGLip FVIII formulation in preclinical models improved *in vivo* pharmacokinetic parameters and increased circulation half-life (29, 37). However, it is unlikely that the prolongation of the circulating half-life is the sole reason for the increased hemostatic efficacy. The PEGLip formulation may increase therapeutic proteins’ effectiveness via several different mechanisms. The presence of platelets is vital for enhancing the hemostatic characteristics of the PEGLip FVIII formulation, according to previous *in vitro* studies evaluating clot formation and lysis by rotating thrombelastography (25, 31). In the clotting process, platelets are crucial. Consequently, it is easier to understand how the PEGLip formulation increases hemostatic effectiveness when considering PEGLip attaches to platelets. Further clots developed when PEGLip-FVIII was introduced to highly hemophilic whole blood or platelet-rich plasma compared to regular FVIII. The aggregates were also more dense and fibrinolysis-resistant. *Ex vivo* rotational thromboelastometry investigations on entire blood from hemophiliac mice demonstrated that mice injected with PEGLip-FVIII had quicker coagulation times than mice injected with free protein (28). Platelets were essential for these kinetics enhancements, coagulation strength, and resistance to fibrinolysis. Since they could not be observed when experiments were conducted on plasma devoid of platelets (38).

**Figure 6:**
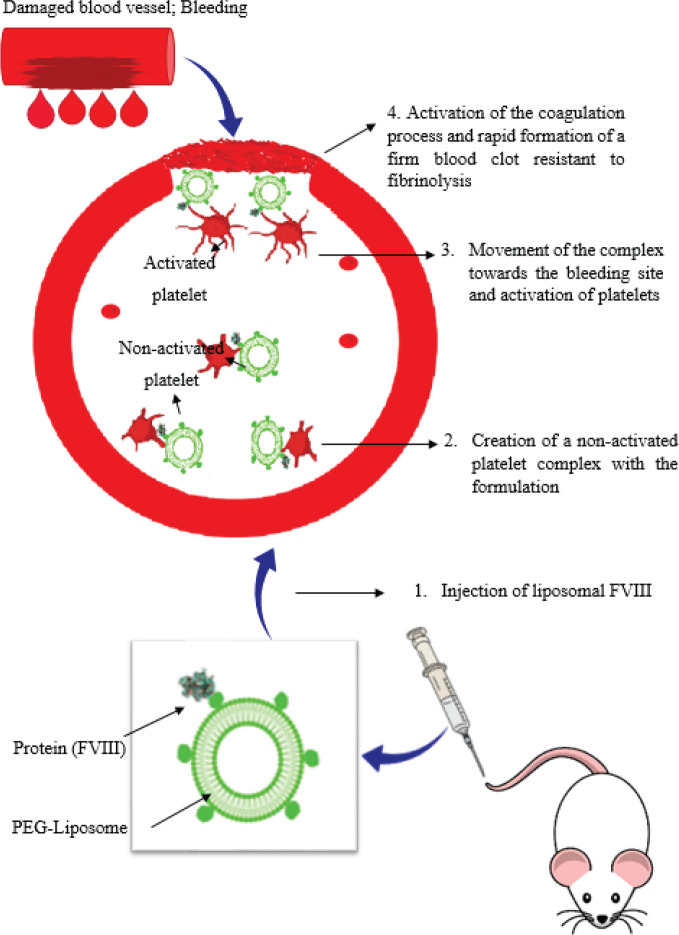
Proposed mechanism of function of liposomal FVIII

Overall, the aggregated outcomes demonstrate the following procedure (Figure 6): during the formulation of liposomal factor VIII, proteins are attached to the surface of pegylated nanoliposomes through covalent bonds. Liposomes attach to inactive platelets when the PEGLip-FVIII formulation is injected into the vessel, creating the platelet/PEGLip/FVIII complex in circulation (39). Because platelets are already enriched with pegylated liposomes carrying factor VIII, they are much more efficient than platelets alone. At the location of a wound, platelets become activated and undergo coagulation cascade formation. The coagulation cascade works more effectively when FVIII is already on the platelets before activation. Clots develop more quickly and are more durable. Even if there is limited circulating FVIII, the interaction of PEGLIP-FVIII with platelets may cause an increase in FVIII at the wound site; thus, although FVIII secretion was only 1% of normal levels, expression of FVIII in platelets efficiently enhanced hemostasis. This could be caused by platelet recruitment to the injury site and local FVIII release from activated platelets (40, 41). 

**Table 1:** Physicochemical characteristics of liposomal formulations and liposomal FVIII (n = 3, mean±SEM)

Formulation	Molar ratio (%)	Z average (nm)	Polydispersity	Surface charge (mV)	Total phospholipid concentrations (mM)		AE% ^a^
					Expected	Observed	
F1-a HSPC/DSPE-mPEG2000	97:3	98.09 ± 2.1	0.141 ± 0.01	-6.71 ± 0.1	120	119.7 ± 0.2	-
F1-b (HSPC/DSPE-mPEG2000)- rFVIII	-	98.48 ± 1.8	0.145 ± 0.01	-6.11 ± 0.1	-	-	87.27 ± 0.1
F2-a HSPC/DSPE-mPEG2000	95:5	99.35 ± 2.03	0.150 ± 0.02	-6.66 ± 0.1	120	119.5 ± 0.3	-
F2-b (HSPC/DSPE-mPEG2000)- rFVIII	-	99.91 ± 1.7	0.158 ± 0.02	-6.01 ± 0.1	-	-	87.09 ± 0.1
F3-a POPC/DSPE-mPEG2000	97:3	87.54 ± 1.9	0.10 ± 0.01	-6.85 ± 0.1	120	119.9 ± 0.1	-
F3-b (POPC/DSPE-mPEG2000)- rFVIII	-	87.91 ± 1.3	0.11 ± 0.01	-5.96 ± 0.1	-	-	86.76 ± 0.1
F4-a POPC/DSPE-mPEG2000	95:5	97.76 ± 1.85	0.128 ± 0.02	-6.99 ± 0.1	120	119.8 ± 0.1	-
F4-b (POPC/DSPE-mPEG2000)- rFVIII	-	98.34 ± 2.1	0.125 ± 0.02	-6.05 ± 0.1	-	-	86.42 ± 0.1

^a ^Attachment efficiency

**Table 2:** Stability studies nanoliposomes stored at 4 °C during time (n = 3, mean ± SD)

	Time (week)	**F1** **-** **HSPC/DSPE-mPEG2000 (97:3)**	**F2** **-** **HSPC/DSPE-mPEG2000 (95:5)**	**F3** **-** **POPC/DSPE-mPEG2000 (97:3)**	**F4** **-** **POPC/DSPE-mPEG2000 (95:5)**
**Z Average (nm)**	0	98.09 ± 2.1	99.35 ± 2.03	87.54 ± 2.1	97.76 ± 2.01
	2	98.13 ± 1.53	99.58 ± 1.24	87.58 ± 2.02	97.80 ± 1.32
	4	98.82 ± 1.87	99.69 ± 1.94	87.59 ± 1.97	97.83 ± 1.87
	8	100.1 ± 2.1	99.78 ± 1.8	88.14 ± 2.03	98.95 ± 1.95
	16	99.88 ± 1.83	97.34 ± 1.92	88.30 ± 1.94	98.01 ± 2.01
	24	102.4 ± 1.85	99.02 ± 2.02	88.02 ± 1.97	98.13 ± 1.96
	36	104.6 ± 2.1	100.42 ± 1.8	88.91 ± 2.2	98.20 ± 1.94
**PDI**	0	0.141 ± 0.01	0.150 ± 0.02	0.1 ± 0.01	0.128 ± 0.02
	2	0.142 ± 0.01	0.141 ± 0.02	0.101 ± 0.02	0.130 ± 0.01
	4	0.144 ± 0.01	0.150 ± 0.02	0.101 ± 0.01	0.129 ± 0.01
	8	0.153 ± 0.02	0.168 ± 0.01	0.121 ± 0.01	0.128 ± 0.01
	16	0.148 ± 0.01	0.153 ± 0.01	0.123 ± 0.01	0.125 ± 0.02
	24	0.143 ± 0.01	0.169 ± 0.02	0.125 ± 0.02	0.127 ± 0.01
	36	0.153± 0.01	0.148 ± 0.02	0.124 ± 0.01	0.128 ± 0.01
**Zeta potential (mV)**	0	-6.71 ± 0.1	-6.66 ± 0.1	-6.85 ± 0.1	-6.99 ± 0.1
	2	-6.48 ± 0.1	-6.60 ± 0.05	-6.98 ± 0.1	-6.85 ± 0.1
	4	-7.14 ± 0.2	-6.95 ± 0.2	-7.49 ± 0.1	-7.83 ± 0.2
	8	-7.81 ± 0.2	-7.11 ± 0.1	-7.81 ± 0.1	-7.07 ± 0.2
	16	-7.62 ± 0.2	-7.65 ± 0.1	-7.93 ± 0.2	-7.54 ± 0.2
	24	-7.99 ± 0.2	-7.90 ± 0.1	-7.45 ± 0.2	-8.16 ± 0.2
	36	-8.66 ± 0.1	-8.62 ± 0.1	-8.76 ± 0.2	-8.84 ± 0.1

## Conclusion

The creation of longer-acting FVIII molecules offers the most potential for prolonging the lives of people with hemophilia A until a specific and definitive treatment is discovered. Over the last several years, research into these compounds has advanced quickly. Among other methods, PEGylated nanoliposomes have been utilized to improve the pharmacodynamics of protein medicines. In this study, we have demonstrated that the PEGLip/rFVIII formulation offers notable advantages compared to conventional rFVIII. FVIII levels were demonstrated to be greater several hours after PEGLip-rFVIII treatment because it persisted in the bloodstream for a more extended period than conventional rFVIII. Currently, using PEGylated liposomes with a high Tm represents a double accomplishment. PEGLip/rFVIII is projected to provide longer bleeding protection and significantly improved survival. According to preclinical findings, liposomal FVIII may enable hemophiliac A patients to benefit from a prophylactic therapy plan with less frequent infusions. It is predicted that PEGLip-FVIII will be a popular therapeutic option for those with hemophilia A and hemophilia with inhibitors.

## Authors’ Contributions

S MR, MR J, and M K designed the experiments; M K performed experiments and collected data; SMR, MR J, SA M, and A H discussed the results and strategy; MR J supervised, directed, and managed the study; S MR, MR J, SA M, A H, and M K approved the final version to be published.

## Conflicts of Interest

 The authors declare that they have no known competing financial interests or personal relationships that could have appeared to influence the work reported in this paper.
